# Data issues in the life sciences

**DOI:** 10.3897/zookeys.150.1766

**Published:** 2011-11-28

**Authors:** Anne E. Thessen, David J. Patterson

**Affiliations:** 1 Center for Library and Informatics, Marine Biological Laboratory, 7 MBL Street, Woods Hole, MA 02543 USA

**Keywords:** life science, informatics, data issues, standards, incentives, escience

## Abstract

We review technical and sociological issues facing the Life Sciences as they transform into more data-centric disciplines - the “Big New Biology”. Three major challenges are: 1) lack of comprehensive standards; 2) lack of incentives for individual scientists to share data; 3) lack of appropriate infrastructure and support. Technological advances with standards, bandwidth, distributed computing, exemplar successes, and a strong presence in the emerging world of Linked Open Data are sufficient to conclude that technical issues will be overcome in the foreseeable future. While motivated to have a shared open infrastructure and data pool, and pressured by funding agencies in move in this direction, the sociological issues determine progress. Major sociological issues include our lack of understanding of the heterogeneous data cultures within Life Sciences, and the impediments to progress include a lack of incentives to build appropriate infrastructures into projects and institutions or to encourage scientists to make data openly available.

## Introduction

The urgent need to understand complex, global phenomena, the data deluge arising from new technologies, and improved data management are driving an agenda to extend the Life Sciences with more data-driven discovery dimensions (National Academy of Sciences 2009). The agenda requires new attitudes, facilities and approaches to sharing and querying existing data (Hey et al. 2009; Kelling et al. 2009). This document addresses some of the more proximate issues that some of the Life Sciences face as they progress towards this “Big New Biology”.

Data-driven discovery refers to hypothesis-testing and the discovery of scientific insights through the novel management and analysis of pre-existing data. It relies on access to and reuse of data which will most likely have been generated to address other scientific problems. While still hypothesis-based, data-driven discovery contrasts with the more familiar process of scientific inquiry based on collecting new data - whether by experimentation or by making new observations. It introduces opportunities to address questions that demand a “scale” of data that cannot be acquired within a single project. It is cost-effective (Piwowar et al. 2011). Data-driven discovery is not new to biology, it is already part of exploring long term trends and is an integral part of the molecular field, but it is not the norm in most sub-disciplines. It requires a large open pool of data across the full breadth of the Life Sciences and into adjacent disciplines. The pool will probably be virtual, with tools accessing data from many repositories. Such a pool will allow biology to join the other “Big” (= data-centric) sciences such as astronomy and high-energy particle physics (Hey et al. 2009). Access to a pool will invite “New” logic, strategies and tools (a “macroscope”) to discover those trends, associations, discontinuities, and exceptions that reveal aspects of the underlying biology which are unlikely to emerge from more reductionist approaches (De Rosnay 1975; Ausubel 2009; National Academy of Sciences 2009; Patterson et al. 2010; Sirovich et al. 2010). An additional benefit is that a pool, and the resources from which it is macerated, may reveal factors not intrinsic to biology which improve our acuity or introduce distortions into knowledge; that is, it can lead to a better understanding of scientific certainty (Evans and Foster 2011).

The emergence of a data-centric Big New Biology is not guaranteed. Current practices in much of the discipline are parochial, with data being generated by individuals or small teams, being called upon to develop insights that are communicated in a narrative style in scientific publications. These small sciences rarely have a formal data culture, data are rarely collected with reuse in mind, they may be discarded, although more recently some journals and some sub-disciplines retain publication-related subsets of data (White et al. 2008). Data sharing requires a stable and effective cyberinfrastructure and the enthusiastic participation of the scientific community (National Science Foundation 2003, 2006; Burton and Treloar 2009; European Science Foundation 2006; http://www.gloriad.org). Registries and repositories must grow to meet the challenges of making data discoverable and accessible. The emerging “Knowledge Organization Systems” (Morris 2010) need to effectively aggregate disparate data sets in part through evolving schemas that define categories of data across the Life Sciences and through ontologies that will intelligently model existing knowledge. Semantic web technologies are needed to achieve flexibility of reuse. Enhanced user interfaces with organizational, analytical and visualization tools will be needed to allow scientists to interact with the data and associated infrastructure. Most existing environments for data management are limited in scope, and need to be improved. The enthusiastic participation of professional biologists requires a readiness to make data available for reuse, and to take advantage of new opportunities in their quest for understanding. The resulting new mesh of biological, computer and information sciences, as well as changes to current cultures, is envisioned as having the capacity achieve the data-centric architecture capable of building new bridges among the sub-disciplines of the Life Sciences and making biology big.

This document reviews technical and sociological issues for biologists in the light of this futuristic vision for the Life Sciences. Many elements, such as data trust and data types have technological and sociological components and in such cases we have combined them for clarity.

## What is meant by data

The term “data” is not used consistently. For some it is limited to raw data, for others the term widens to include any kind of information or process that leads to insights. We prefer to limit the term to neutral, objective, raw data that are largely independent of context, analysis or observer. As data become constrained, filtered and selected, they acquire or are assigned a meaning in the context of what they apply to. This is part of the process that transforms data into information (Ackoff 1989). There is no clear point of transition.

### Contextual categorization of data

The context in which biological data are acquired or generated is important to understanding how data can be appropriately reused. A context may be formed if observers select or interpret their records, because of the limitations of tools or instruments used, or because data are gathered in an unnatural setting such as an experiment or “in silico”. Individuals and technologies are selective and capture a limited subset of all available data. Data are affected by choice of instrument and analytical processes. Some context can be represented through the addition of appropriate metadata to data. We categorize the following broad types of data reflecting the context of their origins.

**A. Observational data** relate to an object or event actually or potentially witnessed by an agent. An agent may be a person, team, project, initiative; and they may call upon tools and instruments. Scientists need to take responsibility to add metadata to the observational data, ideally identifying the agent, date, location, and contexts such as experimental conditions if relevant or the equipment used. Within the Life Sciences, metadata should include taxon names, the basis for identification and/or pointers to reference (voucher) material.

***1. Descriptive data*** are non-experimental data collected through observations of nature. Ideally, descriptive data can be reduced to values about a specified aspect of a taxon, system, or process. Each value will be unique, having been made at one place, at one time, by one agent. Observations may be confirmed but not replicated such that it is important to preserve these data. Preservation often does not occur as data of this type are discarded after completion of the research narrative - the publication. The OBOE project offers a formal framework for descriptive data (Madin et al. 2007a).

Descriptive data can be collected by instruments or by individuals. Data collected by individuals may not represent the world completely or accurately. Mistakes can be made, such as misidentification of taxa (MacLeod et al. 2010). Researchers may be selective about the data they seek to gather, either intentionally or unintentionally, such that data sets have limited applicability. Some individuals may discard data that are not in keeping with their expectations. Few or no raw data may be recorded, such that the information may only be available in an interpreted form. Descriptive data contribute to the “long tail” of small data sets, and often are not well suited to reuse.

**2. *Experimental data*** are obtained when a scientist changes or constrains the conditions under which the expression of a phenomenon occurs. Experiments can be conducted across a broad range of scales - from electrophysiological investigations of sub millisecond processes within cells (Bunin et al. 2005) to manipulations of oceanic ecosystems (Coale et al. 2004). The intent is to dissect the elements of the phenomenon by changing conditions to uncover causal relationships, or to identify variant and invariant elements of biological processes. The raw data that are produced are contextualized by the experimental framework, and may have limited or no value in other contexts. It is important for associated metadata to include information about source and storage of material before the experiment, experimental conditions, equipment, controls and treatments.

**B. Processed data** are obtained through a reworking, recombination, or analysis of raw data. There are two primary types.

***1. Computed data*** result from a reworking of data to make them more meaningful or to normalize them. In ecology, productivity or the extent of the ecosystem are rarely measured directly. Rather they are computed using information or data from other sources to generate measurements of the amount of carbon or mass that is generated per unit area per unit time. While computed data may be held in the same regard as raw data, choices or errors in formulae or algorithms may diminish or invalidate the data created. The raw data that were used and information on how computed data were derived (provenance) are important for reproducibility. The metadata should provide this information. As computed data will grow as the virtual data pool expands, it will be helpful for sub-disciplines to develop appropriate protocols and advertize best practices.

***2. Simulation data*** are generated by combining mathematical or computational models with raw data. Often models seek to make predictions of processes, such as the future distribution of cane toads in Australia under various climatic projections. The proximity of predictions to subsequent observations is used to test the concepts on which the model is based and to improve the model and our associated understanding of biology. Metadata differ dramatically from other data types in that date of the run, initial conditions of the model, resolution of the model output, time step, etc. are important. Rerunning the model may require preservation of initial conditions, model software, and even the operating system (Shirky 2005). Simulation data become less useful as they age and can become a storage burden.

## Sociological issues

As the study of human social behavior, sociology includes the study of the behavior and practices of scientists. If we are to promote a shift to a Big New Biology, we need to understand current data cultures to determine which elements favor a transformation, and which will hinder it.

### 1. Data cultures

The phrase “data culture” refers to the explicit and implicit data practices and expectations that determine the destiny of data. It relates to the social conventions of acquisition, curation, preservation, sharing, and reuse of data. If the goal is to make data digital, standardized and openly accessible in a reusable format, then current data cultures provide starting points to determine the changes that will be needed before that vision can be realized. While a comprehensive survey has yet to be undertaken, it is clear that there is no single data culture for the Life Sciences (Norris et al. 2008; Gargouri et al. 2010; Key Perspectives Ltd 2010; Feijen 2011). This is unsurprising given that Life Sciences range in scope and scale from the field biologist whose data are captured in short-lived notebooks as a prelude to a narrative explanation of observations to the molecular biologist whose data are born digital in near terabyte quantities and are widely shared through global data repositories.

### 2. Readying data for reuse

The preparation of data for reuse in a shared pool often involves a series of steps or stages that relate to the capture, digitization, structure, storage, curation, discoverability, access, and mobility of data. The situation with molecular data achieved by the International Nucleotide Sequence Database Collaboration comprising the DNA Data Bank of Japan (DDBJ), the European Molecular Biology Laboratory (EMBL), and the NCBI GenBank in the USA is exemplary (http://www.insdc.org/). Molecular data tend to be born digital, and are submitted in standard formats to centralized repositories in which they are freely available for reuse in a standard form. A rich diversity of tools, services and applications has evolved to analyze and visualize the data.

**Figure 1.**
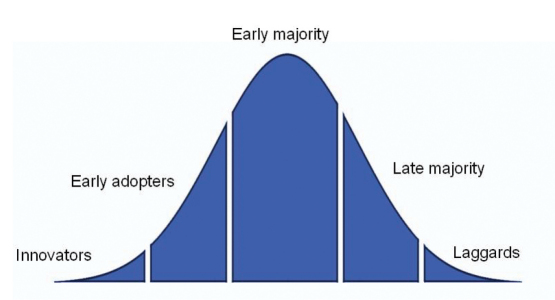
Rogers adoption curve describes the acceptance of a new technology. Life Sciences is still in the Early Adopters phase for accepting principles of data readiness.

Yet, set in the context of Rogers adoption curve (Rogers 1983; Fig. 1), and as suggested by Harnad (2010), Life Sciences, generally, are closer to the early adopters stage of transition to data sharing than other sciences. It is still unusual for data created in most sub-disciplines to be made ready and openly available for sharing (Davis 2009). For these sub-disciplines to join Big New Biology, data practices must change to improve retention of data, their conversion to digital form and placement within schemes of widely agreed standards, and visibility and accessibility with few or no restrictions. The technical aspects of these practices are described in the technical issues section.

### 3. Agents

The term “agent” refers to individuals, groups or organizations - each influencing data cultures.

**Scientists.** As major producers and consumers of Life Sciences data, scientists are important participants in Big New Biology. Within the US there are almost 100,000 biologists (excluding agriculture and health sciences) working outside of academia (United States Department of Labor). The number within academia can be estimated from data on the approximately 2,500 colleges and universities (http://www.globalcomputing.com/american-universities.htm) that employ almost 300,000 academics in science and engineering, 40% of whom work in the Life Sciences (National Science Board 2010a). US research and development endeavors account for approximately one-third of the global effort (National Science Board 2010b). Consequently, changing data practices will directly or indirectly affect as many as 200,000 life scientists in the US and about half a million professionals worldwide (PARSE 2009).

As personal computers and Internet access have become integral components of biological research (Stein 2008), scientists’ views and practices of data sharing have changed. Biologists are increasingly publishing data through repositories like GenBank (http://www.ncbi.nlm.nih.gov/genbank/), their own web sites, or are participating in collaborative environments such as those that allow data to be annotated (e.g. EcoliWiki, http://ecoliwiki.net/colipedia/index.php/Welcome_to_EcoliWiki or DNA Subway for genome annotation, http://dnasubway.iplantcollaborative.org/) or to capture field data using services such as provided by Artportalen (http://www.artportalen.se/default.asp) or eBird (www://ebird.org). An increasing number of databases are providing web services to mobilize data and new tools for visualizing data (e.g. GeoPhyloBuilder, https://www.nescent.org/sites/evoviz/GeoPhyloBuilder, Kidd and Liu 2008). Data processing and management pipelines such as Kepler (https://kepler-project.org/) and VisTrails (http://www.vistrails.org/index.php/Main_Page) are emerging. Yet, for these changes to dominate across the breadth of the discipline and influence the full life cycle of the data, researchers must feel comfortable with design and performance of software systems (Stein 2008). There must be good dialog between the biologists and computer programmers for new tools to be adopted (Lee et al. 2006). Increasingly, biologists will need to be trained in computer and information science (Stein 2008) and include archiving machine-readable data and appropriate metadata as part of their normal workflow (Whitlock 2011). Computer scientists, software engineers, and others who produce code need to develop sensitivity to biology and biological thinking if they are to provide tools that delight life scientists.

Scientists, especially those associated with small science, will need to be more engaged in mobilization of data than at present (Froese et al. 2003, Heidorn 2008, Costello 2009, Smith 2009). Many scientists do share specific data sets with close colleagues (Science staff editorial 2011), yet are insufficiently incentivized to share their data openly. In part, they perceive the risks of making data available as outweighing the rewards (Porter and Callahan 1994, Key Perspectives Ltd 2010). This is despite the fact that papers with openly available data gain more citations (Piwowar et al. 2007). While there are communal repositories for sub-disciplines other than molecular, such as Global Biodiversity Information Facility and Ocean Biogeographic Information System for occurrences data, the majority of sub-disciplines lack appropriate communal repositories.

**Publishers.**Publishers of scientific journals are increasingly involved in data management (Whitlock et al. 2010). Publishers may provide the same services for data that they provide for manuscripts (i.e. peer review, citability, etc. Vision 2010). Some journals require deposition of data as a condition of publication. An example is the joint data archiving policy (JDAP, http://datadryad.org/jdap). JDAP has grown from its original consortium of evolution and ecology journals to include more than a dozen journals (Vision 2010). Dryad (http://datadryad.org/; White et al. 2008), GenBank (http://www.ncbi.nlm.nih.gov/genbank/; Bilofsky and Christian 1988), Protein Data Bank (http://www.wwpdb.org; Berman et al. 2006) and TAIR (http://www.arabidopsis.org/; Rhee et al. 2003) are examples of repositories that benefit from deposition requirements from publishers. Publishers historically controlled the dissemination of the narrative. Some limit access to articles while others, such as PLoS (http://www.plosbiology.org/static/help.action#xmlContent)and Pensoft (http://www.pensoft.net/journals.php) have moved to an open-access model. Although some publishers (http://www.articleofthefuture.com/, Ziegler et al. 2011) are experimenting with enhanced publication to allow researchers to share data sets, illustrations and audio files, we may presume that a publisher-driven model for data sharing is likely to incur charges for access to or submission of data. Many scientists feel this is inappropriate (Key Perspectives Ltd 2010). A model is offered by Thomson Reuters BIOSIS that indexes more than half a million Life Sciences abstracts yearly (http://thomsonreuters.com/content/science/pdf/BIOSIS_Factsheet.pdf). They are compiling metadata such as organism names and Enzyme Commission numbers that can be used to discover sources, and the publisher charges for its discovery services.

**Funding agencies.**Funding agencies worldwide have been called upon to finance informatics research and to promote tools and digital libraries that will underpin the shift towards a Big New Biology paradigm (Hey et al. 2009; National Academy of Sciences 2009). Funding agencies are accountable to the public and to the government (e.g. Coburn 2011). Data cost money and the reuse of data represents a better return for each research dollar invested (Piwowar et al. 2011). In recognition of the importance of data sharing to their investment, funding agencies are increasingly imposing data-sharing requirements on their researchers (Table 1). Yet, many funding agencies, especially outside the US and Europe, do not have data policies or plans to make data available. Of those that do, many require scientists to submit data management plans as a part of their proposals. The plans are designed to explain where data will be deposited, under what terms data may be accessed, and what standards will be used. Many agencies believe in open access to data at the end of a project and have specific timelines for data release. They often acknowledge that the data provider will have a period of exclusive “right of first use” of data.

**Table 1.** List of funding agencies and characteristics of their data policies

**Funding Agency**	**Country**	**Policy**	**Data Management Plan**	**Deposit**	**Standards Compliant**	**Attribution**	**Local Archive**	**Open Source**	**QA/QC**	**Confidentiality**	**IPR/Licensing**	**Metadata Deposit**	**Provides Data for Free**	**Free Access to Publications**	**Notes**
Gordon and Betty Moore Foundation	US	http://moore.org/docs/GBMF_Data%20Sharing%20Philosophy%20and%20Plan.pdf	×			×					×				
Genome Canada	Canada	http://www.genomecanada.ca/medias/PDF/EN/DataReleaseand ResourceSharingPolicy.pdf	×	×	×	×		×	×	×					Data must be made available no later than the publication date or the date the patent has been filed (which ever comes first) at the end of the project
National Institutes of Health	US	http://grants.nih.gov/grants/policy/data_sharing/	×							×					Applies to projects requesting > $500,000, data must be released no later than the acceptance of publication of the main findings from the final data set
Biotechnology and Biological Sciences Research Council	UK	http://www.bbsrc.ac.uk/publications/policy/data_sharing_policy.html	×	×	×						×	×			data release no later than publication or within 3 years of generation, Researchers are expected to ensure data availability for 10 years after completion of project
Natural Environment Research Council	UK	http://www.nerc.ac.uk/research/sites/data/policy.asp	×	×		×					×		×		Data must be made available within 2 years from the end of data collection
Wellcome Trust	UK	http://www.welcome.ac.uk/About-us/Policy/Policy-and-position-statements/WTX035043.htm	×			×									
Department of Energy	US	http://genomicsgtl.energy.gov/datasharing	×	×	×	×	×	×		×		×			Requires deposit of 1) protocols 2) raw data 3) other relevant materials no later than 3 months after publication
Chinese Academy of Sciences	China	http://english.cas.cn/													Requires deposit or no further funding
Australian Research Council	Australia	http://www.arc.gov.au/default.htm													No policy
National Science Foundation	US		×												
Austrian Science Fund	Austria	http://www.fwf.ac.at/en/public_relations/oai/index.html		×										×	Data must be available no more than 2 years after end of project
NASA	US	http://science.nasa.gov/earth-science/earth-science-data/data-information-policy/						×		×					Data can be embargoed for 2 years
NOAA	US	http://www.ncdc.noaa.gov/oa/about/open-access-climate-data-policy.pdf		×									×		
Council for Scientific and Industrial Research	India	http://rdpp.csir.res.in/csir_acsir/Home.aspx													Plan being developed in 2010
North Pacific Research Board	US	http://www.nprb.org/projects/metadata.html		×								×			Data must be transferred to NPRB by the end of the project
Japan Science and Technology Agency	Japan	http://www.jst.go.jp/EN/index.html													None
National Research Foundation	South Africa	http://www.nrf.ac.za/													None

**Governments.**The realization of a Big New Biology will require significant investment in and reorganization of technical and human infrastructure, the creation of new agencies, new policies and implementation frameworks, as well as national and transnational coordination. The scale of these developments will require governmental and intergovernmental participation. Issues that require high-level attention are illustrated by the OECD report that established GBIF (OECD 1999). GBIF has now about 60 national participants and influences national agendas. Especially relevant is the commitment to data sharing with its Suwon declaration (http://www2.gbif.org/SignedSUWONdeclaration_small.pdf). This underscores the importance of data sharing to science, conservation and sustainability. INSDC, which collates the sharing of molecular data via the US-based NCBI Genbank, the European EMBL, and the Japanese DDBJ, is another example of international informatics initiatives in the Life Sciences (http://www.insdc.org/policy.html).

Several countries have established governmental digital data environments inclusive of the data.gov environments (http://www.data.gov/, http://data.australia.gov.au/, data.gov.uk), or more specialist agencies such as Conabio in Mexico (http://www.conabio.gob.mx/), ABRS, ERIN and ALA in Australia (http://www.environment.gov.au/biodiversity/abrs/, http://www.environment.gov.au/erin/, http://www.ala.org.au/), ITIS in US (http://www.itis.gov/) or the European Environment Agency (http://www.eea.europa.eu/data-and-maps).

In respect to the economics at this level, OECD, when establishing GBIF, compared the cost of the molecular informatics infrastructure (millions of dollars) against the benefits to pharmaceutical, health and agricultural businesses worth billions of dollars (OECD 1999). The costs of international cooperation on biodiversity informatics must be set against the estimated economic value of the world’s natural capital of tens of trillions (millions of millions) of dollars (Costanza et al. 1997; TEEB 2010). The OECD estimates costs of sustaining infrastructure to be 25% of the costs of generating raw data. Yet, an allocation of as little as 5% of research funding could provide billions of dollars for data preservation (Schofield et al. 2010).

**Universities.**With in excess of 20,000 universities (and institutions modeled on Universities) worldwide (Webometrics Ranking of World Universities; http://www.webometrics.info/methodology.html), employing an estimated 5–10 million academics and associated researchers, universities form the largest research and development initiative. Collectively, Universities are a significant source of new data and given their international communal character, will be important as consumers of the data pool. The support, infrastructure and services that Universities provide will be a major determinant of the flow and fate of data. Some environments, such as the SURF foundation (http://www.surffoundation.nl/en/actueel/Pages/Researchersenhancetheirpublications.aspx) seek to unite research institutes through the application of new technologies. SURF serves the Dutch context and currently emphasizes 5 disciplines; Life Sciences are not included.

Universities may or may not regard themselves as owners (having IP rights) of data and so may regulate access to data generated in-house or as part of collaborative projects. Universities may or may not have policies that require the retention of research data for a limited period usually in the range of 3 to 7 years. The University of Melbourne policy is based on guidelines from the National Health and Medical Research Council/Australian Vice Chancellors’ Committee and specifies that “Data must be recorded in a durable and appropriately referenced form” for a minimum of 5 years (http://www.unimelb.edu.au/records/research.html). The Chinese University of Hong Kong encourages researchers to deposit their data in the University Service Center upon completion of their research (http://www.usc.cuhk.edu.hk/Eng/SharingPolicy.aspx). US universities are bound to comply with the requirements of OMB Circular A-110 (Uniform Administrative Requirements for grants and agreements with Institutions of Higher Education, Hospitals, and Other Non-Profit Organizations – http://www.whitehouse.gov/omb/circulars_a110). This specifies that financial records, supporting documents, statistics, and all other records produced in connection with a financial award, including laboratory data and primary data *are to be retained by the* *institution* for a specified period. OMB A-110 also states “The Federal awarding agency(ies) reserve a royalty-free, nonexclusive and irrevocable right to reproduce, publish, or otherwise use the work for Federal purposes, and to authorize others to do so.” Many universities have data policies that target administrative data and administrative agenda rather than on promoting the use of data for academic purposes (e.g. “(This) University must retain research data in sufficient detail and for an adequate period of time to enable appropriate responses to questions about accuracy, authenticity, primacy and compliance with laws and regulations governing the conduct of the research” – http://ora.ra.cwru.edu/University_Policy_On_Custody_Of_Research_Data.pdf). As their policies improve, Universities will need to play a significant role in educating staff and students as to the value of data. They will be the focus of reshaping the skill base on which the Big New Biology will rely (Doom et al. 2002). New trans-discipline curricula will ensure that biologists gain informatics skills and that computer scientists develop sensitivity to the challenges and needs in Biology.

**Museums and herbaria.**Museums and herbaria play special roles within the Life Sciences. Along with libraries, they have a mandate for the long-term preservation of materials. Those materials include several billion specimens of plants, animals and fossils collected by biologists over 3 centuries (Chapman 2005a; OECD 1999; Vollmar et al. 2010). Those collections provide invaluable information as to changing distributions of species, provide access to extinct species, and inform research into defining species. They have special value in some phenomena that motivate the agenda for Big New Biology, such as distribution of invasive species, consequences of deforestation, and so on. Chapman (2005a) provides an exhaustive treatment of potential and actual value of primary biodiversity records.

**Citizen scientists.**Citizen scientists are non-professionals who participate in scientific activities. The appealing richness of nature, its accessibility, and our reliance on natural resources ensures that biology attracts an especially high participation by the citizenry (Silvertown 2009). The academic skills of citizen scientists cover a massive spectrum, from those with casual interests in nature or science to individuals who publish in the scientific literature. The tens of millions of birders in the US (Kerlinger 1993) translates to more than 100 million worldwide. The number of recreational fishermen in marine waters approaches that of birdwatchers (Arlinghaus and Cooke 2009; Cisneros-Montemayor and Sumaila 2010), and an estimated 500 million people have livelihoods attached to fishing (ftp://ftp.fao.org/FI/brochure/climate_change/policy_brief.pdf). That suggests that the potential citizen scientist community exceeds 1 billion people. This remarkable pool can be called upon to add the “sightings” (occurrence of a given species at a particular location at a particular time) which can be used to monitor the changing distributions and abundances of endemic and invasive species. The Swedish ArtPortalen (http://www.artportalen.se/default.asp) has in 10 years compiled more than 26 million sightings at a rate of about 10,000 per day, illustrating the irreplaceable role of the citizen scientist. Several mobile phone apps exist that allow naturalists to record species occurrences in the field (BirdsEye from eBird, http://www.getbirdseye.com/ and Observer from WildObs, http://wildobs.com/about/observer). Data on occurrences, or of the first occurrences of flowering or appearance of migratory species, can be called on to test scientific hypotheses as to the impact of climate change on the biosphere. Citizen scientists are significant monitors of endangered species – providing the first evidence that some presumed-extinct species, such as the coelocanth (http://www.extinctanimal.com/the_coelacanth.htm), Wollemi pine (http://www.wolganvalley.com/pdf/wolgan-valley/en/media-centre/fact-sheets/Wolgan%20Valley%20Wollemi%20Pine%20Fact%20Sheet.pdf?1=6), ivory-billed woodpecker (http://www.cryptomundo.com/cryptozoo-news/ibw-rainsong/), Lord Howe Island stick insect (http://www.kidcyber.com.au/topics/Lordhowestick.htm) and mountain pygmy possum (http://animaldiversity.ummz.umich.edu/site/accounts/information/Burramys_parvus.html) are still with us.

**Repositories*.***A repository provides services for management and dissemination of data inclusive of, ideally, making data discoverable, providing access, protecting the integrity of the data, ensuring long term preservation and migrating to new technologies (Lynch 2003). Most repositories typically handle a specific data type at a particular granularity. Thousands of repositories already exist for managing Life Sciences data and hold tens of millions of items (Table 2; see Jones et al. 2006, repository66.org and http://datacite.org/repolist for more). However, it is estimated that less than 1% of ecology data is captured in this way (Reichmanet al. 2011).Some sub-disciplines do not have repositories and the volume of data in some fields has led even exemplar repositories such as GenBank to question their capacity to host all data (http://www.ncbi.nlm.nih.gov/About/news/16feb2011; http://phylogenomics.blogspot.com/2011/06/sequenceshort-read-archive-sra-back.html).

**Table 2.** Examples of repositories for Life Sciences data.

**Repository**	**Type of Life Sciences Data**	**location**
AlgaeBase	algae names and references	http://www.algaebase.org/
ArrayExpress	microarray	http://www.ebi.ac.uk/arrayexpress/
Australia National Data Service	general research data	http://www.ands.org.au/
ConceptWiki	concepts	http://conceptwiki.org/index.php/Main%20Page
CSIRO	fisheries catch	http://www.marine.csiro.au/datacentre/
Data.gov	natural resources data	http://www.data.gov/
Diptera database	Dipteran information	http://www.sel.barc.usda.gov/diptera/biosys.htm
EMAGE	gene expression	http://www.emouseatlas.org/emage/
ENA	gene sequences	http://www.ebi.ac.uk/ena/
Ensembl	genomes	http://uswest.ensembl.org/index.html
Euregene	renal genome	http://www.euregene.org/
Eurexpress	transcriptome	http://www.eurexpress.org/ee/
EURODEER	movement of roe deer	http://sites.google.com/site/eurodeerproject/home
FishBase	fish information	http://www.fishbase.org/
GBIF	occurrences	http://www.gbif.org/
GenBank	gene sequences	http://www.ncbi.nlm.nih.gov/genbank/
GEO	microarray	http://www.ncbi.nlm.nih.gov/geo/
GNI	names	http://gni.globalnames.org/
INBIO	Costa Rican biodiversity	http://www.inbio.ac.cr/es/default.html
INSPIRE	spatial	http://inspire.jrc.ec.europa.eu/index.cfm
KEGG	genes	http://www.genome.jp/kegg/
Life Sciences Data Archive NASA	effects of space on humans	http://lsda.jsc.nasa.gov/
MassBank	mass spectra	http://www.massbank.jp/index.html?lang=en
MGI	mouse	http://www.informatics.jax.org/
MorphBank	images	http://www.morphbank.net/
OBIS	occurrences	http://www.iobis.org/
OMIM	human genes and phenotypes	http://www.ncbi.nlm.nih.gov/omim
PDB	molecule structure	http://www.pdb.org/pdb/home/home.do
PRIDE	proteomics	http://www.ebi.ac.uk/pride/
PubMed	citations	http://www.ncbi.nlm.nih.gov/pubmed/
Stanford Microarray Database	microarray	http://smd.stanford.edu/
tair	Arabidopsis molecular biology	http://www.arabidopsis.org/
TOPP	animal tagging	http://www.topp.org/topp_census
TreeBase	phylogenetic trees	http://www.treebase.org/
TROPICOS	plant specimens	http://www.tropicos.org/
UniProt	protein sequence and function	http://www.uniprot.org/
WILDSPACE	life history information	http://wildspace.ec.gc.ca/more-e.html
WRAM	wireless remote animal monitoring	http://www-wram.slu.se/

Repositories range in functionality from basic data stores to collaborative databases that incorporate analysis functions (WRAM, Wireless Remote Animal Monitoring, www-wram.slu.se). Some repositories host heterogeneous data sets (such as oceanographic databases – http://woce.nodc.noaa.gov/wdiu/, http://www.nodc.noaa.gov/, http://www.ices.dk/ocean/), but those that provide normalization, standardization, atomization and quality control services (see below) will facilitate the reuse of data and will play a stronger role in data-intensive science. That many older repositories are difficult to access or are not maintained (Wren and Bateman 2008) reveals the need for appropriate funding and persistence strategies. Repositories can fail as a result of policy shifts, funding instability, management issues, or technical failures (Lynch 2003). Such failures can undermine acceptance of digital scholarly work by the community at large. As data repositories become more important over time, they must be trusted to provide high quality services reliably (Schofield et al. 2010). The trustworthiness of archives can be assessed using criteria catalogues (Klump 2011) available from organizations like the Digital Curation Center (Innocenti et al. 2007) and the International Standards Organization (ISO 2000). The Center for Research Libraries has assembled a list of ten principles for data repositories that addresses administrative and technical concerns (http://www.crl.edu/archiving-preservation/digital-archives/metrics-assessing-and-certifying/core-re).

## Technological issues

The second array of challenges that need to be addressed as we move towards Big New Biology are technical issues that affect the distribution, preservation, accessibility and reuse of data.

### Making data accessible

**Figure 2.**
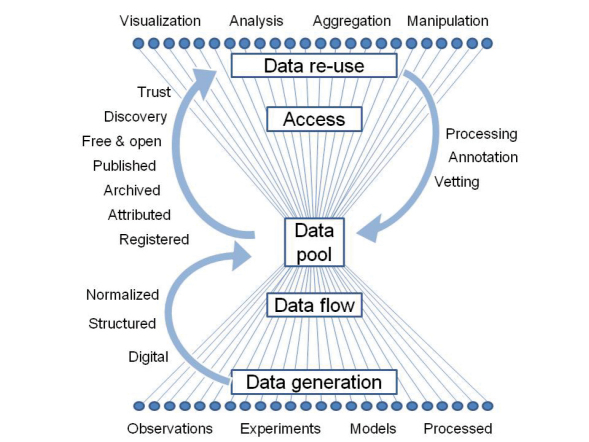
A Big New Biology can only emerge with a framework that optimizes reuse. Ideally, data should be in forms that can flow from source into a common pool and can flow back out to consumers, be subject to quality control, or be enhanced through analysis to rejoin the pool as processed data.

The effective reuse of data requires that an array of conditions (Fig. 2) is optimized.

**Data need to be retained.** Relatively few data acquired historically have been retained in an accessible form by scientists, projects or institutions (Pullin and Salafsky 2010). The culture of disposing of data following publication, termination of a grant, relocation or retirement of a scientist is clearly incompatible with the vision of a data-centric biology. While work practices in some areas, such as those in which data are born digital, or institutions with a strong tradition of preserving records, include data retention or their submission to a repository, much of the small biology lacks such a culture (Key Perspectives Ltd 2010). There is as yet an unresolved debate as to whether all data should be retained, or if subsets of data should be selected for retention, or if retained data should be subject to periodic review for deaccessioning.

**Data need to be digital.** Digitization is a prerequisite for data mobility. Considerable amounts of relevant data are not yet in a digital format (Chavan and Krishnan 2001; Vollmar et al. 2010; Schofield et al. 2010; Heidorn 2008). Non-digital formats include notes, books, photographs and micrographs, papers, and specimens. The Biodiversity Heritage Library and similar projects are now in the process of digitizing some half billion pages of biology text (Gwinn and Rinaldo 2009). Digital metadata about non-digital materials have value as they make the data discoverable and increase incentives for digitization.

**Data need to be structured.** Digital data may be unstructured (e.g. in the form of free text or an image) or they may be structured into categories that are represented consecutively or periodically through the use of a template, spreadsheet or database. The simple structure of a spreadsheet allows records to be represented as rows. Data occur within the cells formed by the intersection of rows and columns defined by metadata (headers). A source may mix both structured and unstructured data such as when fields include free-form text, images, or atomic data. Unstructured data, such as the legacy data to be found in an estimated 500 million pages of text, can be improved through annotation with metadata provided by curators or through tools such as natural language processing tools.

**Data should be normalized.** Normalization brings information contained within different structures to the same format (or structure). Normalization may be as simple as consistently using one type of unit. Placing data within a template is a common first step to normalization. Normalization is a prerequisite for aggregating data. When data are structured and normalized, they can be mobilized in simple formats (tab delimited or comma delimited text files) or can be transformed into other structures to meet agreed upon standards. DiGIR is an early example of a data transformation tool (http://digir.sourceforge.net/). More contemporary tools, such as TAPIR or IPT from GBIF (http://ipt.gbif.org/) can output data in an array of normalized forms.

**Data should be standardized.** Standardization indicates compliance with a widely accepted mode of normalizing. Standards provide terms that define data and relationships among categories of data. Two basic types of standards that are indispensable for management of biological data are metadata and ontologies. Organizations such as TDWG develop new standards, and catalogs of standards and ontologies are available on the web (http://otter.oerc.ox.ac.uk/biosharing/?q=standards, http://wg.sti2.org/semtech-onto/index.php/The_Ontology_Yellow_Pages).

Metadata are terms that define data in ways that may serve different purposes, such as helping people to find data of relevance (that is they aid the discovery of data - Michener 2006), or allow data to be drawn together (federated). Metadata standards define how data should be named and structured, thus reducing the heterogeneity of terms. Standards may mandate the types of metadata that are appropriate for different types of data. Sets of metadata terms agreed upon by a community are referred to as controlled vocabularies, one of the most extensive bearing on the Life Sciences is the Ecological Metadata Language (EML; Fergraus et al. 2005). Scientific names are argued by some as having the potential to act as an extensive system of metadata (Patterson et al. 2010; See discussion below).

By articulating what metadata should be applied and how they should be formatted, standards introduce the consistency that is needed for interoperability and machine reasoning. For example, a marine bacterial RNA sequence collected from the environment ideally might be accompanied by metadata on location (latitude, longitude, depth), environmental parameters, collection metadata (collection event, date of collection, sampling device), and an identifier for the bacterium. Without such metadata, the scope of possible queries is much reduced. Examples of minimum reporting requirements have been established by the MIBBI project (Taylor et al. 2008). Numerous metadata guides are available within Life Sciences (Table 3). There are software programs available to assist in the collection and organization of metadata (such as Morpho, http://knb.ecoinformatics.org/morphoportal.jsp Higgins et al. 2002; Metacat, http://knb.ecoinformatics.org/software/metacat/, Jones et al. 2002; MERMAid, http://www.ncddc.noaa.gov/metadataresource/metadata-tools).

**Table 3.** Examples of standards and their location.

**Standard**	**Location**	**Type**
ABCD	http://www.bgbm.org/TDWG/CODATA/Schema/default.htm	Schema
Bioontology	http://www.bioontology.org/	Ontology Repository
BIRN	http://www.birncommunity.org/	
Cardiac Electrophysiology Ontology	http://bioportal.bioontology.org/ontologies/39038	Ontology
CMECS	Coastal and marine ecological classification standard http://www.csc.noaa.gov/benthic/cmecs/cmecs_doc.pdf	Vocabulary
Comparative Data Analysis ontology	http://sourceforge.net/apps/mediawiki/cdao/index.php?title=Main_Page	Ontology
Darwin Core	http://wiki.tdwg.org/twiki/bin/view/DarwinCore/	Metadata
Dublin Core	http://dublincore.org/	Metadata
Ecological Metdata Language	http://knb.ecoinformatics.org/software/eml/	Metadata
Environment Ontology	http://www.environmentontology.org/	Ontology
Evolution Ontology	http://code.google.com/p/evolution-ontology/	Ontology
Experimental Factor Ontology	http://www.ebi.ac.uk/efo/	Ontology
Federal Geospatial Data Committee	http://www.fgdc.gov/	Metadata
Fungal Anatomy	http://www.yeastgenome.org/fungi/fungal_anatomy_ontology/	Ontology
Gene Ontology	http://www.geneontology.org/	Ontology
Homology Ontology	http://bioportal.bioontology.org/ontologies/42117	Ontology
HUPO	http://www.psidev.info/index.php?q=node/159	Vocabulary
Infectious Disease ontology	http://www.infectiousdiseaseontology.org/Home.html	Ontology
International Standards Organization	http://www.iso.org	Metadata
Marine Metadata Interoperability	http://marinemetadata.org/	Metadata
Miriam	http://www.ebi.ac.uk/miriam/main/datatypes/	Vocabulary
National Biodiversity Information Infrastructure	http://www.nbii.gov/portal/community/Communities/NBII_Home/	Metadata
Ontology of Microbial Phenotypes	http://sourceforge.net/projects/microphenotypes/	Ontology
Open Biological and Biomedical Ontologies	http://www.obofoundry.org/	Ontology Repository
Phenotype Quality Ontology	http://obofoundry.org/wiki/index.php/PATO:Main_Page	Ontology
Plant Ontology	http://www.plantontology.org/	Ontology
SDD	http://wiki.tdwg.org/twiki/bin/view/SDD/Version1dot1	Schema
Species Profile Model	http://wiki.tdwg.org/SPM	Schema
Taxonomic Concept Schema	http://www.tdwg.org/activities/tnc/tcs-schema-repository/	Schema
TDWG	http://www.bgbm.org/TDWG/acc/Referenc.htm	Metadata
Teleost Anatomy Ontology	https://www.phenoscape.org/wiki/Teleost_Anatomy_Ontology	Ontology

Anontology is a formal statement of relationships among concepts represented by metadata terms. Ontologies enable discovery of and reasoning on data through those relationships. Ontologies may use formal descriptive languages to define the relationships. Ontologies are regarded as having great promise (Madin et al. 2007b): “An ontology makes explicit knowledge that is usually diffusely embedded in notebooks, textbooks and journals or just held in academic memories, and therefore represents a formalization of the current state of a field. If ontologies are properly curated over the longer term, they will come to be seen as modern day (albeit terse) textbooks providing online and up-to-date biological expertise for their area. In another sense, they will provide the common standards needed for producing a strong biological framework for integrating data sets. Ontologies therefore provide the formal basis for an integrative approach to biology that complements the traditional deductive methodology” (Bard and Rhee 2004).

Ontologies are part of “Knowledge Organization Systems”. Those relating to biodiversity have been discussed by Morris (Morris 2010). Ontologies contribute to the semantic annotation of data and the artificial intelligence it enables. As an example, a simple search for information about the bird - robin, seeks to match some or all of character string r-o-b-i-n or to character strings in text within a data object or annotating the data object. The system cannot discriminate among data on American robins, European robins, Robin Reliant cars, Robin Wright Penn, or Robin the boy-superhero. However, if the query for “robin” is placed in the context of an ontology, such as one that declares that a context is the Turdidae, an informed system is able to return only relevant results from appropriately annotated data. In addition to more precise searching, ontological structures allow the computer to perform inference, a form of artificial intelligence. For example, an ontology that establishes that turdidae is_a bird and wing is part_of a bird, allows the inference that an American robin has wings and that data on wings, flight, or migrations may be discoverable. Larger interconnected ontologies allow more complex inferences.

Many ontological structures are available for use in Life Sciences (Table 3). Some, such as the observational (http://marinemetadata.org/references/oboeontology, http://www.nceas.ucsb.edu/ecoinfo, https://sonet.ecoinformatics.org/) and taxonomic ontologies (below), have broad applicability - the first within the field of ecoinformatics and the second to biodiversity informatics. Users can adopt existing structures or create their own using an ontology editor such as Protégé (http://protege.stanford.edu/) or OBOEdit (http://oboedit.org/). The search engines, Swoogle (http://swoogle.umbc.edu/) and Sindice (http://sindice.com/), search over 10,000 ontologies and can return a list of those that contain a term of interest. Services such as these help users to determine if an existing ontology will meet his/her needs. Often, a user may need to use parts of existing ontologies or merge several ontologies into a single new one. Defining relationships between terms in different ontologies can be accomplished through the use of automated alignment tools such as SAMBO and KitAMO (Lambrix and Tan 2008). The development and integration of ontologies is best carried out using formal languages (such as OWL, http://www.w3.org/TR/owl-ref/) and by individuals versed in their logical foundations. The Biodiversity Information Standards (TDWG) organization (http://www.nhm.ac.uk/hosted_sites/tdwg/first_minutes.pdf) and GBIF have been prime movers in developing organizational frameworks for biodiversity information. Unfortunately, there are competing systems of standards and not all aspects of biology have established standards. Various efforts are under way to create broad scope ontologies (http://www.loa-cnr.it/index.html, http://www.tonesproject.org/, http://www.geneontology.org/). The promise of ontologies is as yet not fully realized as “The semantic web is littered with ontologies lacking ... data” (Joel Sachs, pers. comm.).

The system of latinized binomial names (such as *Homo sapiens*) introduced for species in the mid-18th century by Linnaeus is an extensive system of potential metadata for data management in the Life Sciences. They have been used to annotate virtually every statement about any of our current catalog of 2.2 million living and extinct forms of life (Raup 1991, Chapman 2009) until quite recently. Now they are being supplemented with molecular identifiers, but at this time they are well suited to form the basis of a names-based cyberinfrastructure for Biology (Patterson et al. 2008, 2010). This approach has been used for life-wide, data organization projects such as the Encyclopedia of Life (http://www.eol.org/). Placement of names within hierarchical classifications offers ontological frameworks that enable data aggregation, drilling down through data sets, and browsing through data. The conversion of names into a formal ontology has been explored through projects such as ETHAN (http://spire.umbc.edu/ont/ethan.php). Our current understanding of biodiversity and the system of names is maintained by a specialist group of 5,000–10,000 professional taxonomists worldwide (Hopkins and Freckleton 2002), who generally are unaware of the informatics potential of names as a near universal indexing system for biological data. The Global Names Architecture is a new global initiative that links names databases and associated services to deliver names-based services to end users (Patterson et al. 2010).

**Data will need to be atomized.** Atomization refers to the reduction of data to minimal semantic units and stands in contrast to complex data such as images or bodies of text. In atomized forms, data may exist as numerical values of variables (e.g. “length of tail: 5.3 cm”), binary statements (e.g. “chloroplasts: absent”), or as the association with metadata terms from agreed upon vocabularies (e.g. “part of lodicules of lower floret of pedicellate spikelet of tassel”; *Zea mays* ontology ID ZEA:0015118, http://bioportal.bioontology.org/visualize/3294). Atomized data on the same subject can be brought together if the data are classified in a standard way. Atomization is necessary for machine-based analysis of data from one or more datasets. Many older data centers capture data as files (or packages of files) and the responsibility for extraction of data atoms falls to the user. This can be time consuming suggesting that, in the future, atomization needs to occur at or near the source of raw data, becoming part of the responsibilities of the author of the data, the software in which data are logged, or data centers that can provide services to transform data sets.

**Data need to be published.** Projects participating in a Big New Biology will increasingly make data visible and accessible (i.e. published). Scientists may publish data by displaying them in unstructured or structured formats on local, project, or institutional web sites; or they may seek to place data in central repositories. In science generally, over three-quarters of the published data are in local repositories (Science staff editorial 2011) which can provide few guarantees of persistence (see “Data are Archived” below). In such environments, the responsibilities for discovery of data, negotiations with copyright holders and acquisition of data lie with the consumer. This is time consuming and unlikely to be done on a large scale. Publication is better served through the use of central, domain-specific repositories because they are more likely to persist, provide better services, and offer the framework around which third-parties develop value-adding services. The molecular data environment consortium of ISNDC is a good example of this model. Only a small fraction of data are deposited in such environments (less than 10% of the science community generally - Science staff editorial 2011), with costs and absence of an organizational framework (metadata and archiving environments) being cited as reasons.

Publication of atomized data is essential for large scale data reuse. Data must be able to move from one computer to another in an intelligent way. As illustrated by the Global Biodiversity Information Facility (http://www.gbif.org/informatics/standards-and-tools/using-data/web-services/), scientific initiatives can add RSS feeds, web services, and APIs (Application Programming Interfaces) to their web sites to broadcast new data or to respond to requests for data. An API facilitates interaction between computers in the same way that a user interface facilitates interactions between humans and computers. Without such services, data may need to be screen scraped from the web site, a process that is usually costly (because the solution for each site will differ) and, at worst, may require manual re-entry of data. A service-oriented approach is scalable but incurs overhead. They are probably best served through community repositories that can call on appropriate domain-specific knowledge.

**Data must be archived.** It is preferable that data, once published, are persistent (Feijen 2011). Projects, initiatives and host institutions have little incentive to preserve data for the long term as the process incurs a cost, and repositories that emerge within projects may have limited life spans (e.g. OBIS, http://www.iobis.org/). However, data archiving can be viewed as a good investment by funding agencies (Piwowar et al. 2011). Central repositories that are not dependent on short-term funding are better positioned to archive data making them persistent. The three global molecular databases that make up the International Nucleotide Sequence Database Collaboration provide an excellent example of how domain-specific repositories may operate. Because they are not funded through short-term projects, and because they mirror each other, such repositories guarantee the persistence of data, and empower scientists to develop projects that involve substantial analyses of shared data (Tittensor et al. 2010). Persistence can be assisted by institutions such as libraries and museums that specialize in the preservation of artifacts or by governmental intervention (the US-based National Institutes of Health support GenBank). An alternative solution to persistence is an effective business model that allows a data center to be sustained by income from services that it sells; or by providing essential services that ensure support from the community of users. Examples of commercial models include the Chemical Abstracts Service of the American Chemical Society (www.cas.org/) or Thomson Reuters’ Zoological Record (http://thomsonreuters.com/products_services/science/science_products/a-z/zoological_record/).

**Data will ideally be free and open.** Open Access, the principle of providing unconstrained access to information on the web, improves the uptake, usage, application and impact of research output (Harnad 2008). Open Access has been applied widely to the process of publication, where it is seen as an alternative to the model in which publishers act as gatekeepers. Open Access has been applied less to data, and while this extension is natural, it is not straightforward (Vision 2010). Attitudes about sharing data freely within Life Sciences vary broadly. In sub-disciplines like genomics, data sharing is the norm with some researchers sharing their data immediately via blogs or wikis (http://www.carlboettiger.info/research/lab-notebook and http://pathogenomics.bham.ac.uk/blog/). Communities that value data sharing may have no formal recognition for such activities nor supportive technical infrastructure. Other communities have a strong sense of data ownership and are antagonistic to open data sharing. Researchers in these communities expect to be directly involved in any further analyses of their data. Databanks for these communities often require registration and/or a fee to gain access. Some data may be regarded as too sensitive to be made fully accessible (Key Perspectives Ltd 2010).

**Data can be trusted.** Once data are accessed, consumers may reveal errors and/or omissions. Biological data can be very dirty, especially if they were acquired without expectation that they would be shared later. Any data cleaning procedures should be documented to aid the consumer in assessing whether the source is “suitable for their purpose” (Chapman 2005b). The creation of “quality loops” allow comments to flow back to the source where data can be annotated or modified, and returned to users for renewed vetting. Webhooks (http://iphylo.blogspot.com/2011/02/web-hooks-and-openurl-making-databases.html) offer a mechanism to exploit APIs to have comments returned to source. Any editing of data can lead to the undesirable outcome that variant forms of the same data may coexist. To some extent, versioning of data sets can be used to discriminate between modified datasets, but users need to cite the version used in analyses (Zhang et al. 2007).

**Data must be attributed.** Scientists gain credit in part through attribution. The permanent association of identifiers with open data offers a means of linking attribution to the data and of tracking reuse (Cryer et al. 2009). The association of authors’ names with data motivates contributions (or lack of credit demotivates them). Attribution favors the development of quality loops to correct errors or otherwise comment on the data. Special care is needed when attributing data resulting from the combination of one or more existing sets so that all intellectual investment is properly credited. Dryad, a JDAP partner, provides data citations through the use of DataCite DOIs with an unrestrictive Creative Commons Zero license, thus promoting clear citation and reuse of data (Vision 2010). Community norms can ensure proper attribution of CC0-licensed data (Fauchart and von Hippel 2008). The Panton Principles provide guidelines for licensing data (http://pantonprinciples.org/).

**Data can be manipulated.** A value of having large amounts of appropriately annotated data available on the web is that users can explore, in addition to search for, data. Data exploration may result from a desire to test a hypothesis. It is therefore desirable to have tools that draw data together, analyze or visualize them. Exploratory systems include: Humboldt (Kobilarov and Dickinson 2008) which operates like a faceted filter for Linked Data; Parallax which accesses data in Freebase and has the ability to interact with data on multiple web pages at once (Huynh and Karger 2009); and Microsoft Pivot (http://www.getpivot.com/) allows a user to interact with large amounts of data from multiple Internet sources.

Visualizations have the capacity to reveal patterns, discontinuities and exceptions that can inform us as to underlying biological processes, appropriateness of data sets, or consistency of experimental protocols. Visualizations can be used to display results with analyses of large data sets. Through visualizations we may help address the challenge stated by Fox and Hendler (2011) that “... many of the major scientific problems facing our world are becoming critically linked to the interdependence and interrelatedness of data from multiple instruments, fields and sources”. The absence of effective visualization is creating a bottleneck within data-intensive sciences (Fox and Hendler 2011). Solutions need to be found in relatively simple low end visualizations (as wonderfully catalogued in http://www.visual-literacy.org/periodic_table/periodic_table.html) to high end tools designed for the data deluge that themselves may call on graphics and visualization standards to be pipelined into rich, complex, and flexible aids. Many Life Sciences data sets can be drawn together and visualized using the geospatial element such as with LifeMapper (http://www.lifemapper.org/) or by OBIS and GBIF (inter alia; Webb et al. 2010). Geospatial metadata, along with temporal, publication, and names metadata are especially valuable as integrators of diverse data sets.

**Data need to be registered and discoverable.**Registries index data resources to alert potential users to their availability. Search engines, the normal indexers of web-accessible materials, are not good at revealing database contents - only about half of the open data in repositories are indexed by search engines (McCown et al. 2006). Discovery is made possible by the addition of coarse grained discovery metadata. Registry functions need to expose discovery metadata to make data sets more visible. As an example, GBIF provides registry level service for biodiversity data (http://www.gbif.org/informatics/standards-and-tools/integrating-data/resource-discovery/). Registries that cover software (http://en.bio-soft.net/geshi.html, http://www.equisetites.de/palbot/software/software.html) or web services (www.biocatalogue.org) are valuable in promoting awareness of tools for data capture, conversion and processing. Successful domain repositories, such as GenBank, have well-structured and detailed metadata that enable detailed search and enhanced discoverability. In the absence of such registries, researchers turn to peers, publications or the thousands of minor data sets available via the Internet. Under these circumstances, it is hard to know when, or if, all relevant data are found. There is a need for a broad-spectrum registry and indexing service (like a Google for data) where researchers can post pointers to their own data, search for desired data and have a means to quickly preview the results. Examples of this exist in Europe with OpenDOAR (http://www.opendoar.org/) and in India with Database of Biological Database (http://www.biodbs.info/), each with thousands of listings. Semantic annotation of data greatly increases discoverability, and is discussed below.

### The semantic web and Big New Biology

The “semantic web” has many definitions, but here we think of it as a technical framework that promotes automated sharing and reuse of data across disciplines (Campbell and MacNeill 2010). The semantic approach has advantages of being flexible, evolvable, and additive. A semantic infrastructure will lead to machine-mediated answers to more complex queries than previously possible (Stein 2008). The foundations for automated reasoning lie in the annotation of data with agreed metadata, linked through a network of ontologies, and queried using conventions (languages) such as RDF, OWL, SKOS and SPARQL (Campbell and MacNeill 2010). The mass of appropriately annotated data that can be accessed through the Internet is referred to as LOD (Linked Open Data). Through common metadata, the data can be linked to form a Linked Open Data cloud. At this time, Life Sciences makes up 9% of the triples in LOD and 51% of the links (Bizer et al. 2011).

Berners-Lee has promoted four guidelines for linked data (Berners-Lee et al. 2006):

1. The use of a standard system of Uniform Resource Identifiers (URIs) as “names” for things

2. The use of HTTP URIs so that the names can be looked up on the internet and the data accessed

3. When a URI is looked up, it should return useful information using standards (RDF, SPARQL)

4. Links to other URIs so that users can discover more things.

A URI is a type of persistent identifier made up of a string of characters that unambiguously (at least in an ideal world, see Booth 2010 for discussion) represents data or metadata and can be used by machines to access the data. Different data sets can be linked when they refer to the same URIs. For example, several marine data sets could be linked because they identify the same investigator, sampling event, or location. The most useful classes of terms that are likely to serve the needs of the Life Sciences are georeferences (which can link data from the same location held in different repositories), names of taxa (the common denominator to the majority of statements about biodiversity), publications and identities of people that can be interconnected through devices such as FOAF (friend-of-a-friend) to find collaborators, relevant data, as well as to dig into the world of scientific literature, the latter being linkable through devices such as DOIs to show citation trends, influential publications, etc. (Patterson et al. 2010).

RDF is a language that defines relationships between things. Relationships in RDF are usually made in three parts (often called triples), Entity:Attribute:Value. A machine-readable form in RDF may be a statement that “American robin:has_color:red”. Each term is ideally defined stringently by controlled vocabularies and ontologies, and each part represented within the triple as a URI. The “Value” can be a URI or a literal - the actual value. An advantage of RDF is that it allows datasets to be merged, for example TaxonConcept and Wikipedia (http://www.slideshare.net/pjdwi/biodiversity-informatics-on-the-semantic-web). A goal of the Linking Open Data project is to promote a data commons by registering sets in RDF. As of March 2011, the project had grown to 28 billion triples and 395 million RDF links (Bizer et al. 2011). The EU project, Linking Open Data 2, received €6.5 million to expand Linked Data by building tools and developing standards (http://lod2.eu/Welcome.html).

Transformation of data from printed narrative or spreadsheet to semantic-web formats is a significant challenge. Based on existing ontologies, there is enough information to create 10^14^ triples in biomedicine alone (Mons and Velterop 2009). At the time of writing, this quantity far exceeds the capacity of any system to process the information.

Life Sciences stand to benefit greatly from the advantages of linked data (Reichman et al. 2011), but need additional investment in mechanisms that ensure quality, provenance and attribution. Provenance identifies sources and, among other things, can ensure attribution and be part of quality control processes. Several software packages currently exist for tracking provenance (such as Kepler, https://kepler-project.org/; Taverna, http://www.taverna.org.uk/; VisTrails, http://www.vistrails.org/index.php/Main_Page). Bechhofer et al. (2010) advocate the use of Research Objects (ROs) as a mechanism to capture additional value necessary to make the semantic web work for science. Provenance of ROs would satisfy recent calls for “open science” that argue that not only data should be open, but so should be associated methods and analyses (Reichman et al. 2011).

Semanticization enables nanopublication, a form of publication that extends traditional narrative publication (Groth et al. 2010) and allows attribution to be associated with the semantic web (Mons and Veltrop 2009). Nanopublications relate to publication of triples. A uniquely identifiable triple is a statement. A triple with a statement for a subject is called an annotation and a set of annotations that refer to the same statement is called a nanopublication. The annotations add attribution and context to the statement. The concept is not widely accepted.

## Discussion

A Big New Biology holds much promise as a means to address some large proximate scientific challenges. Macroscopic tools will enable discovery of hidden features and better descriptions of relationships within the complexity of the biosphere. Yet, to date, progress towards the vision varies enormously from the successes with high-throughput biology to virtual stasis in some small science biology. Considerable effort is needed to catalog current practices, and to define the sociological transformations that will be required to improve the likelihood of success. If the transformation is to be purposeful, then it will need general oversight, discipline-specific reviews, and a description of the actual and desirable components of the Knowledge Organizational System for Biology and their relationships. Some obvious challenges relate to standards and associated ontologies, incentivizing participation, and assembling an appropriate infrastructure and skill base.

**Standards and Ontologies.**Data standards bring order to the virtual data pool on which a Big New Biology will rely. While complex and finely grained metadata are needed for analyses and for the world of Linked Open Data, the first challenge is to improve the discoverability of data. This process has traditionally been supported by word-of-mouth at conferences or in publications. With standards, registries can enable users to find data sets containing information about taxa, parameters, times, processes, or places of interest. If metadata are absent or incomplete, then the data sets cannot be discovered or reused and cannot contribute to Big New Biology.

Automated data discovery, aggregation and analysis require more comprehensive standards than those currently available for many of the Life Sciences. Instead of a comprehensive system of standards, there is a piecemeal system of metadata, vocabularies, thesauri, ontologies, and data transfer schemas that overlap, compete, and have gaps. Greatest progress is being made outside the Life Sciences (such as georeferencing), or in high-investment areas where data are born digital (such as in genomics, Taylor et al. 2008). Given the richness of biodiversity and interactions, a comprehensive system of standards will necessarily be extremely complex, and be costly to implement. This creates a tension: whether to promote the comprehensive annotation of data with a significant overhead that deters participation versus pursuing a more minimalistic annotation that can set a grander process in motion. As the commitment to standards is not widespread, the minimalistic approach is more likely to gain traction. The perspective that “The semantic web is littered with ontologies lacking ... data” noted above warns us against starting with complex structures. Metadata and their inter-relationships will need a framework that is designed to allow initial discipline-specific standards to become more finely grained and for the parts to merge into a dynamic grand schema. The world of Linked Open Data provides a good model for this, but given that few data are appropriately annotated, it has yet to realize its potential.

Two organizational frameworks for Life Sciences data are as yet under-exploited. The first is the system of georeferencing that is in use in rich applications in earth sciences, cartography, and so on. Information on occurrences of species is compiled in central databases such as GBIF and OBIS, has been and is being collected in vast quantities by a myriad of citizen scientists. Its potential is well illustrated by some large-scale applications such as the impressive charting of bird migrations (Marris 2010), meta-analyses of oceanic biota (Webb et al. 2010), or web sites that emphasize locally relevant biota (http://zipcodezoo.com/). Less well developed, but arguably with more potential for many sub-disciplines of the Life Sciences, is the transformation of taxonomic and phylogenetic knowledge into an information management system that uses Latin names and molecular identifiers as metadata and classifications and phylogenies as ontological frameworks for the metadata (Patterson et al. 2010).

**Incentives.**Despite widespread calls for scientists to make data more widely available, this has yet to happen for many sub-disciplines (Dittert et al. 2001, Harnad 2008, Mandavilli 2011, Piwowar 2011). Only about 10% of data make their way to open repositories (Savage and Vickers 2009, Science staff editorial 2011). A current impediment to data sharing is that the benefits derived are often greater for the consumer than the producer (Porter and Callahan 1994). Other reasons are the lack of resources, infrastructure, and incentives for sharing. Sociological, financial, legal and technical barriers must be surpassed for communities to become directly involved in populating and maintaining data pools, a requisite for success and scalability (Feijen 2011).

In surveys, (Froese et al. 2003, Kohnke et al. 2005, RIN 2008, Costello 2009), scientists give the following five reasons not to share data. The first relates to intellectual property: A scientist’s funding and professional recognition relies on receipt of credit for work done. Until scientists receive credit for data publication, there will be little motivation to redirect efforts from more rewarding activities (such as exploring nature or writing papers) towards data mobilization. This problem can be solved with an infrastructure capable of creating citations for data and tracking data use (Froese et al. 2003). The second relates tolegal and confidentiality issues as some data cannot be shared, such as data concerning people (Guttmacher et al. 2009) or location of endangered species (Froese et al. 2003), proprietary information, or because employers or funders claim that they have copyright over data. The infrastructure must have mechanisms to protect necessary confidentiality. Some data can be anonymised, and in the case of endangered taxa, protection can be accomplished by fuzzing data, so that exact locations or identities are obscured (Froese et al. 2003). Thirdly, there is concern over misuse or misinterpretation of data, which, once in the literature, cannot be unpublished. This is not a new problem, but it will increase as data producers lose control and can no longer act as “gate-keepers”. Part of the solution lies in developing stringent metadata and format standards such that data are released only when there are sufficient metadata to ensure that all users understand the context and limitations of the data. Until such time, disclaimers can alert consumers about inappropriate reuse (Froese et al. 2003, Smithsonian 2011). Fourthly, scientists are concerned that publication can expose errors in their data or weaknesses of analysis. Errors may include insufficient, inaccurate or inappropriate data encoding, metadata, or analysis. Third parties may reveal the selective or inappropriate use of data to emphasize particular arguments. Given the noisy and rich nature of biology, there can be no such thing as a perfect data set; all are incomplete. Errors or gaps uncovered by subsequent users can be dealt with openly and honestly, thereby enhancing the body of scientific data. Finally, there is the issue of sustainability. Project-based data repositories run a risk of being abandoned at the end of the funding cycle. This increases doubts that data curation activities are a good use of resources. It is cheaper to curate data properly than it is to gather it again (Heidorn 2008, Piwowar et al. 2011), and some data, such as data on past distributions of species, are irreplaceable and thus priceless. From an economic perspective, persistent discipline-specific repositories are attractive. There are considerable academic benefits from engaging with repositories. Scientists who share data often report increased book and/or photograph sales, increased web site hits and higher visibility for their projects (Froese et al. 2003). There is greater citation impact for open-access articles (Gargouri et al. 2010). In larger consortia, scientists (such as those studying phylogenetic relationships) who pool data are able to answer questions they could not answer if they were limited to the data that they themselves generated. Some publishers are incentivizing early data-sharing by granting an embargo to the data producers (Kaye et al. 2009) to alleviate fears of being “scooped” (Reichman et al. 2011). An emphasis on “carrots” such as these may be much more effective means of promoting data-sharing than the “sticks” (in the form of funding agency requirements, Kaye et al. 2009; Table 1).

**Figure 3.**
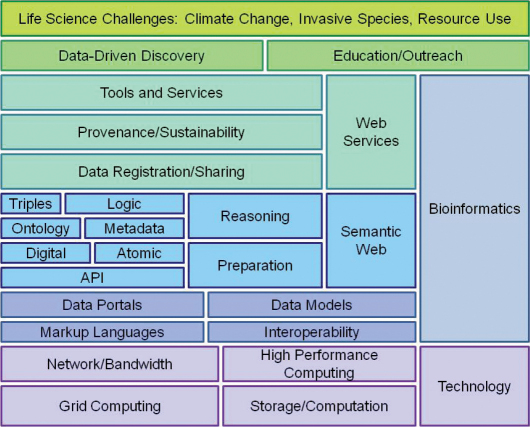
Technical infrastructure needed for Big New Biology to fully emerge (based on Sinha et al. 2010).

**Infrastructure.**In addition to challenges to incentivize scientists in the direction of data-sharing, the infrastructure for a Big New Biology is incomplete. Funding agencies, like the National Science Foundation in the US, require projects to have plans for data management - a requirement that presumes data persistence. The infrastructure needed to guarantee persistence will require an investment well beyond the usual 3–5 year funding cycle into multi-decadal periods and coordination that has international dimensions. The infrastructure must include tools to capture data, policies, data standards, data identifiers, registration of discovery-level metadata, and APIs to share data (Fig. 3). There is as yet no index of data-sharing services (for some initial steps see datacatalogs.org and DataCite http://www.datacite.org/repolist) nor a framework in which such elements could be integrated. There is little assessment of which elements of data plans will lead to persistence of data or their reuse. In the absence of these elements, principle investigators are left to make their own policies, use their own systems, and to finance the processes. As long as the response is piecemeal, there can be no assurances of interoperability, efficiency or persistence. At this time, research scientists need to be supported by data managers and data archivists. Institutional libraries and museums are well placed to shift their agendas to include data management and the preservation of digital artifacts and so may fill this gap, providing institutional, regional or discipline-based services. It is hoped that the ongoing NSF Data Net projects can contribute significantly to the infrastructure.

A new technical challenge is the lack of bandwidth to distribute data from modern data-intense technologies. The problem is illustrated by high throughput molecular biology with tera and petabyte scale data sets (Cochrane et al. 2009). Proposed solutions include Bio-Mirror (http://www.bio-mirror.net/) which consists of several servers holding the same data, or the Tranche Project (https://trancheproject.org/), which shares repository functions across servers. The latter has a high administrative overhead. Peer-to-peer sharing systems such as BitTorrent (Langille and Eisen 2010) overcome potential bandwidth problems by sharing data sets without a central repository. Users of BioTorrents benefit from lower bandwidth use, faster transfer times and data publication. Although terabit per second line rates are on the horizon (Hillerkuss et al. 2011), bandwidth problems are likely to persist as part of the interplay between the evolution of new data-generating instruments and the limitations of the infrastructure to make data freely available to all. We may expect to see a growth of specialist centers that will offer analysis, visualization, and data transformation services on behalf of the users.

## Conclusion

There is growing pressure from scientists, funding agencies and governments to use new information technologies to effectively manage the increasingly vast amounts of data emerging from new technologies, to integrate these with smaller data sets, and to enhance the communal nature of science. If successful, biology will be enriched with data-intensive dimensions better suited to address large scale and trans-discipline problems. The transition requires many technical advances and cultural changes. Progress on the technical front to date clearly demonstrates that technical issues can be resolved. The process of sociological adaptation is less convincing. Some sub-disciplines (molecular domains) have embraced data-intensive dimensions, some (environmental ecology) are in transition, and others (such as taxonomy) are just beginning. A much better understanding of the existing cultures is needed before we can promote solutions that will realign the traditions of each community with the common goal of shared data use. Training environments such as Universities need to create a new cadre of scientists trained in computer sciences and biology. Other pressing challenges to data integration relate to the development of comprehensive and agreed metadata and ontologies, and to the semanticization of data so that the discipline can take advantage of the Linked Open Data cloud. The long tail of small data sets presents a special challenge - that of bringing heterogeneous data sets together. At this time, the common denominators that are likely to be effective are georeferencing, citations, and names. All require further investment. None of the elements of the transition will come quickly or cheaply, but these transformations are needed if we are to make the Life Sciences less parochial and more capable of responding to major research challenges.
